# Overlooking undernutrition? Using a composite index of anthropometric failure to assess how underweight misses and misleads the assessment of undernutrition in young children

**DOI:** 10.1016/j.socscimed.2008.01.021

**Published:** 2008-05

**Authors:** Shailen Nandy, J. Jaime Miranda

**Affiliations:** a University of Bristol, School for Policy Studies, 8 Priory Road, Bristol BS8 1TZ, United Kingdom; b Department of Epidemiology and Population Health, London School of Hygiene and Tropical Medicine, London, United Kingdom; c School of Public Health and Administration, Universidad Peruana Cayetano Heredia, Lima, Peru

**Keywords:** Undernutrition, Indicators, Developing countries, Millennium Development Goals, Young children

## Abstract

In light of current international interest in the Millennium Development Goals, this short report examines the suitability of one of the primary indicators being used to assess progress. Using anthropometric data on 46,784 children aged 0–35 months in seven developing countries, we show how the weight for age (underweight) indicator is problematic but not for the reasons conventionally cited. We show that the information it provides about the degree and direction of change in undernutrition over time sometimes contradicts that provided by other conventional indicators. We demonstrate the potential of an alternative indicator, the composite index of anthropometric failure (CIAF), which can be used to show the overall extent of undernutrition among children, and which provides an unequivocal statement on the direction and degree of change in undernutrition over time. Given the fundamental importance of undernutrition to child survival and health, the issues raised will be of interest to and have implications for policy makers and planners alike.

## Introduction

Undernutrition is acknowledged to play a major role in the premature death of millions of children in developing countries (Black, Morris, & Bryce, 2003). Those it does not kill, it renders vulnerable to infection and disease, blighting the lives of hundreds of millions (WHO & World Bank, 2002; World Bank, 1993). In addition to the eradication of extreme poverty, the eradication of hunger is a primary Millennium Development Goal (MDG) with progress assessed using the proportion of underweight children (i.e. those whose weight for their age is below a set threshold) as an indicator (United Nations Statistics Division, 2006). Recently, however, concern about whether this and other Millennium Development Goals will be met has been raised (Lawn, Costello, Mwansambo, & Osrin, 2007), given resources and global attention have been diverted to addressing other issues like the so-called War on Terror and wars in Iraq and Afghanistan.

The choice of underweight as an indicator is an interesting one, not least because (a) it refers primarily to the nutritional status (a related but different concept to hunger) of young children, and (b) it tells us nothing about hunger or food deprivation among adults or older children. If one accepts child underweight as a proxy indicator for both hunger and hunger among the wider population, there remains another problem. Underweight is but one of a number of indicators using anthropometric data (i.e. heights and weights) to assess nutritional status. Children experiencing stunting – low height for their age – and wasting – low weight for their height – are also considered undernourished, with the former reflecting chronic or longer term undernutrition, and the latter acute or more recent undernutrition (WHO, 1995). Underweight is used as a composite of stunting and wasting, but does not distinguish between the two. However, in a population, there will be different degrees of overlap between each indicator – i.e. some underweight children will also experience stunting and/or wasting, some stunted children might not be underweight, and some children might simultaneously experience all three forms of anthropometric failure – stunting, wasting and underweight. As a result none of these conventional indicators used on their own can truly reflect the *overall* burden of undernutrition.

An alternative indicator – the composite index of anthropometric failure (CIAF) – has been proposed (Svedberg, 2000) and used successfully (Nandy, Irving, Gordon, Subramanian, & Davey Smith, 2005). This short report addresses some of the issues it raises and its potentially interesting implications.

## Data and methods

Demographic and health survey (DHS) data (ORCMacro, 2006) for seven countries are used to compare the CIAF with conventional indicators of stunting, wasting and underweight. The countries are selected merely for purposes of illustration. The DHS are cross-sectional, nationally representative, standardised surveys used regularly by international organisations like the WHO and UNICEF to assess child health and nutrition (Vaessen, 1996; Wirth et al., 2006). Rates of undernutrition according to each indicator were calculated from z-scores provided for children aged 0–35 months. Children whose z-scores for each indicator fall below −2 standard deviations of the original WHO/NCHS reference population median are conventionally classified as stunted, wasted or underweight (WHO, 1995). In response to concerns about the suitability of the WHO/NCHS reference population for assessing the nutritional status of children in developing countries (de Onis, Garca, & Habicht, 1997), the WHO has recently introduced a new, more internationally representative reference population (Garza & de Onis, 2004; de Onis, Onyango, Borghi, Garza, Yang, & for the WHO Multicentre Growth Reference Study Group, 2006). Estimates of undernutrition presented in this report are based on the original NCHS/WHO reference population.

Table 1 provides details on the countries included in the study, the survey year and the sample size i.e. number of children aged 0–35 months for whom anthropometric data were available.

More detailed information on the construction of the CIAF is reported elsewhere (Nandy et al., 2005), but in short, it is a summary index which identifies children who experiences either stunting, wasting or underweight, or any combination of these. The same threshold of minus 2 standard deviations is used. The CIAF can also be used to show the different combinations of anthropometric failure, so for example, it is possible now to know what proportion of children in a population simultaneously experience stunting, wasting and underweight.

## Results

### Prevalence of undernutrition

Table 2 shows the rates of undernutrition in the selected countries. Rates of stunting and wasting are highest in India and Ethiopia and lowest in Bolivia and Peru. Underweight, however, is highest in India and Nepal, and lowest in Bolivia and Peru. The CIAF, which we use to assess overall undernutrition, shows India and Ethiopia have the highest prevalence rates. The difference between the CIAF and underweight is more than 10% points in almost every case. This suggests underweight as an indicator clearly misses numbers of undernourished children.

### Changes in undernutrition

As one of the aims of the first MDG is to reduce by half the proportion of people suffering from hunger (United Nations Statistics Division, 2006), what we need to know are the relative changes from one time period to another. Table 3 presents estimates of undernutrition from consecutive DHS for three of the seven countries with data, as well as the relative changes. Stunting and wasting both appear to decrease in India and Peru, but increase in Zimbabwe. Underweight behaves differently, and falls in all three countries suggesting an improvement in nutritional status and contradicting the information provided by stunting and wasting. The CIAF, which takes account of all three indicators, shows a fall in overall undernutrition in India and Peru, but an increase in Zimbabwe.

## Discussion

Conventional indicators of undernutrition each provide important information on different aspects of undernutrition (e.g. chronic versus acute). This information is valuable in itself, especially for clinicians and fieldworkers, who need to respond differently to different forms of undernutrition. From a policy and planning perspective, however, it is also important to know the *overall* scale of the problem, so that sufficient resources are allocated. At present, the CIAF is the only indicator able to do this. Information from conventional indicators about changes in the prevalence of undernutrition over time may be contradictory and confusing, as in the case of Zimbabwe (Table 3). The CIAF takes the differences between the three conventional indicators into account, and so is more able to provide an indication of changes in undernutrition. It might also be noted that underweight may be exaggerating the magnitude of change. While underweight in India and Peru suggests falls in relative terms of around 11%, the CIAF shows progress may be more limited, with relative falls of only 6 and 4%, respectively. This raises serious questions about the use of underweight as an indicator for monitoring progress towards the first MDG. A related issue, concerning the introduction of the WHO's new international reference population, also has implications about assessing progress towards the Millennium Development Goals, and researchers will need to ensure they compare like with like. Studies comparing prevalence rates based on the new and old reference populations show interesting differences, with the prevalence of underweight decreasing in children 5 months and older, but the prevalence of stunting and wasting increasing (de Onis et al., 2006).

The CIAF can be used to examine the synergistic impact on health and mortality of different combinations of stunting, wasting and underweight. Children with multiple anthropometric failures are more likely to be ill, and to come from poorer households (Nandy et al., 2005), with those experiencing a triple failure carrying the greatest morbidity (and potentially mortality) risk. Clinicians can use the CIAF to explore the interaction between disease, mortality and different combinations of anthropometric failure. Planners could use the CIAF to identify communities or areas where a large proportion of children experience multiple failures and so allocate resources to areas of greatest need. The CIAF has been applied successfully in India and Kenya (Berger, Hollenbeck, & Fields-Gardner, 2006; Seetharaman, Chacko, Shankar, & Matthew, 2007), and recently in an extensive assessment of child poverty in Tajikistan (Baschieri & Falkingham, 2007).

If current indicators provide mixed messages about both the scale of the problem and degree (and direction) of change, then it is time to consider alternatives. Reducing undernutrition is a stated goal of the international community, and the key to doing this lies in the reduction of poverty and improvement of living conditions. In a world of unparalleled economic and health resources, such a task is not only possible but also a moral imperative. Sadly, given current progress, one suspects this most important of Millennium Development Goals is unlikely to be met by 2015.

## Figures and Tables

**Table 1:** Sample details

Country and DHS year	Number of children aged 0–35 months in sample
Zimbabwe 1999	1589
Tanzania 1999	1673
Bolivia 1998	3452
Nepal 2001	3698
Ethiopia 2000	5905
Peru 2000	6071
India 1998	24,396

Total	46,784

**Table 2:** Prevalence of undernutrition by indicator

Country/year	Stunting (low height for age)		Wasting (low weight for height)		Underweight (low weight for age)		CIAF (overall undernutrition)		Difference between CIAF and underweight
	%	95% CI	%	95% CI	%	95% CI	%	95% CI	
India 1998	45.2	(44.6–45.8)	15.9	(15.4–16.4)	47.1	(46.5–47.7)	59.8	(59.2–60.4)	12.7
Ethiopia 2000	45.0	(43.8–46.3)	13.0	(12.2–13.9)	45.7	(44.4–46.9)	58.0	(56.8–59.3)	12.3
Nepal 2001	43.1	(41.5–44.7)	12.2	(11.1–13.3)	46.8	(45.2–48.5)	56.5	(54.9–58.1)	9.7
Tanzania 1999	38.0	(35.7–40.4)	6.5	(5.3–7.7)	30.4	(28.2–32.6)	45.9	(43.5–48.3)	15.5
Zimbabwe 1999	26.9	(24.7–29.0)	7.2	(6.0–8.5)	14.1	(12.4–15.8)	35.8	(33.4–38.2)	21.7
Bolivia 1998	24.3	(22.9–25.7)	1.9	(1.5–2.4)	9.1	(8.2–10.1)	26.6	(25.1–28.1)	17.5
Peru 2000	22.0	(21.0–23.1)	1.2	(0.9–1.4)	7.8	(7.1–8.4)	23.8	(22.7–24.8)	16.0

**Table 3:** Relative changes in undernutrition over time

Country/year	Stunting (%)	Relative change in stunting (%)	Wasting (%)	Relative change in wasting (%)	Underweight (%)	Relative change in underweight (%)	CIAF (Overall undernutrition) (%)	Relative change in CIAF (%)
India 1992	47.3	−4.4*	19.5	−18.5*	52.7	−10.6*	63.5	−5.8*
India 1998	45.2		15.9		47.1		59.8	

Zimbabwe 1994	21.6	24.5*	5.5	32.7*	15.7	−10.2*	29.0	23.4*
Zimbabwe 1999	26.9		7.3		14.1		35.8	

Peru 1996	22.9	−3.9*	1.5	−20.0*	8.8	−11.4*	24.9	−4.4*
Peru 2000	22.0		1.2		7.8		23.8	

*Chi square significant.
